# ST-Segment Elevation in Conditions of Non-cardiovascular Origin Mimicking an Acute Myocardial Infarction: A Narrative Review

**DOI:** 10.7759/cureus.30868

**Published:** 2022-10-30

**Authors:** Kanishk V Khurana, Aditya Ranjan

**Affiliations:** 1 Medicine and Surgery, Jawaharlal Nehru Medical College, Datta Meghe Institute of Medical Sciences, Wardha, IND; 2 Otolaryngology - Head and Neck Surgery, Jawaharlal Nehru Medical College, Datta Meghe Institute of Medical Sciences, Wardha, IND

**Keywords:** electrocardiogram (ecg), emergency, non-cardiac chest pain, st-segment elevation myocardial infarction (stemi), myocardial infarction

## Abstract

The most widespread presenting ailments among patients visiting the emergency department are chest pain and shortness of breath. These symptoms lead any doctor to a probable diagnosis of myocardial infarction (MI). Detailed patient history, testing of blood samples for cardiac biomarkers that are indicative of cardiovascular necrosis, ultrasound methods, electrocardiography, and coronary computed tomography (CT) could all be beneficial to support the diagnosis. Out of these, electrocardiography is the most important and commonly done investigation in the emergency departments for patients presenting with chest pain and shortness of breath. However, interpreting these patients' electrocardiograms (ECGs) may be a matter of concern and worry. T wave and ST-segment changes are often of interest in the early signs of myocardial ischemia. Despite its incredible sensitivity, ST-segment deviation (elevated or depressed) has a low specificity because it can be seen in a variety of other cardiac and non-cardiac diseases. When ST-segment anomalies are identified, clinicians must consider many additional characteristics (such as risk factors, symptoms, and anamnesis), as well as all other possible diagnoses.

All of these scenarios of patients presenting in the emergency department with chest discomfort and shortness of breath showing ST-segment abnormalities can leave a healthcare professional wondering whether to start treatment for acute myocardial infarction, through either the administration of a fibrinolytic agent, exposing patients to both the benefits and risks of fibrinolysis, or invasive coronary angiography. An astute physician may be able to recognize fabricated differential diagnosis mimicking ST-segment elevation myocardial infarction (STEMI) in some situations. Failure to recognize these imposters can result in inefficient resource utilization, which can expose patients to unjustified risk and increased rather than decreased death and morbidity. Since the danger of cerebral hemorrhage from blood thinners is significant, in patient-care scenarios, in order to rule out percutaneous coronary intervention (PCI), a thorough assessment of the ECG is essential to consider diseases other than acute myocardial infarction, especially the ones that are non-cardiac in origin. The goal of this narrative review is to give an overview of the significant disorders that are non-cardiac in origin that can mimic an ST-segment elevation myocardial infarction (STEMI).

## Introduction and background

An electrocardiogram (ECG) is an electrical trace of the heart, which is a noninvasive diagnostic technique used by physicians to evaluate any cardiovascular diseases, especially myocardial infarction (MI). Since cardiovascular illnesses are the major cause of death, it becomes significant for healthcare personnel to gain skills and expertise in reading ECGs in order to give the best care as soon as possible [1].

When the ST segment on an ECG is elevated, our immediate worry is if the patient is experiencing an ST-segment elevation myocardial infarction (STEMI). However, ST-segment elevation can be caused by a variety of different diseases, even by those that primarily are non-cardiac in origin. However, some clinical features and cardiac biomarkers of myocardial infarction may be present among patients who have ST-segment elevation; hence, this is a real challenge faced by physicians. STEMI occurs when transmural myocardial ischemia causes cardiac muscle damage or necrosis. The ECG criteria for diagnosing STEMI given by the American College of Cardiology, American Heart Association, European Society of Cardiology, and World Heart Federation committee are new ST-segment elevation at the J point in two contiguous leads with a cutoff value greater than 0.1 mV in all leads except V2 and V3. In leads V2-V3, the cutoff value is larger than 0.2 mV in males over 40, greater than 0.25 mV in males under 40, and greater than 0.15 mV in females [2].

The pathophysiological reason behind ST-segment elevation or depression in an ECG is the occlusion of one or more coronary arteries that deliver blood to the heart. The most common cause of this abrupt suspension in blood flow is the rupture of an old plaque, erosion, or the segmentation of coronary arteries, which results in an obstructive clot [3].

Some of the other cardiovascular pathologies that depict ST-segment elevation in an electrocardiogram are as follows: pericarditis, myocarditis, right bundle branch block, stress cardiomyopathy (Takotsubo), early repolarization, acute vasospasm, and left ventricular hypertrophy [2,4].

As discussed above, there are several diseases of non-cardiovascular origin that shows ST-segment elevation, which mimics STEMI in an electrocardiogram. Some of these diseases that are discussed in this narrative review are COVID-19, pulmonary embolism (PE), pneumothorax, lung metastasis, hiatal hernia, small bowel obstruction (SBO), acute pancreatitis, gastric perforation, esophageal rupture, pheochromocytoma, thyrotoxicosis, hypocalcemia, hyperkalemia, and subarachnoid hemorrhage (SAH).

All of these disorders have been discussed thoroughly in this review article according to their respective systems. Our aim with this article is to enhance the current knowledge of physicians about electrocardiograms demonstrating ST-segment elevation in circumstances other than myocardial infarction so that they are able to make a better and more accurate diagnosis in patients that present with complaints of chest pain, shortness of breath, and ECG mimicking an acute myocardial infarction.

## Review

Pulmonary diseases

COVID-19

Severe acute respiratory syndrome coronavirus 2 (SARS-CoV-2) is a viral infectious disease that was first documented in December 2019 in Wuhan, China. In March 2020, the World Health Organization (WHO) declared it a global pandemic. Most patients present with clinical manifestations such as dry cough, fever, dyspnea (difficulty in breathing), headache, anosmia (the loss of olfactory sensations), ageusia (the loss of taste), and pneumonia [5]. COVID-19 causes not only pulmonary distress but also several cardiovascular manifestations in the body, e.g., pericarditis, myocarditis, cardiac arrhythmia, thromboembolism, and heart failure [6]. Thus, it is important to recognize and treat cardiac pathologies such as myocardial infarction in patients with COVID-19 as it is a time-sensitive disorder. These cardiac pathologies, which are associated with COVID-19, can mimic ST-segment elevation myocardial infarction (STEMI) on an electrocardiographic test (ECG), but on coronary angiography, there is no evidence of obstructive pathology [7]. One systematic review of COVID-19 with ST-segment elevation revealed that 83% of patients suffering from COVID-19 had obstructive coronary artery disease (OCAD) on coronary angiography and the remaining 17% of the patients who presented with ST-segment elevation had nonobstructive coronary artery disease, and they underwent invasive coronary angiography, which turned out to be futile [8]. Another case report revealed that COVID-19 was also associated with pericarditis, which mimics ST-segment elevation myocardial Infarction (STEMI), in which elevation was seen in leads Ⅱ, Ⅲ, and augmented vector foot (aVF) on electrocardiography. Troponin levels were also elevated (0.10 ng/ml), so the differential diagnosis formed was acute coronary syndrome (ACS), but after 24 hours, serial ECG showed that it resolved ST-segment elevation leading to a diagnosis of pericarditis and spared invasive coronary catheterization [9].

Pulmonary Embolism

Pulmonary embolism is a congestion of the major artery of the lungs and its branches caused by a blood clot (embolism), most commonly as a result of a deep vein thrombosis (DVT) complication. Due to the blockage of blood flow by the clot, the pulmonary pressure increases, which leads to high pressure in the right ventricles and prompts heart failure by ventricular dysfunction (cor pulmonale) [10]. Due to the variable clinical presentation of pulmonary embolism, it is often misinterpreted as a myocardial infarction because both of them present with similar symptoms of dyspnea, tachypnea, and chest pain [11]. The electrocardiogram often exhibits tachycardia/atrial fibrillation, right bundle branch block, or the suggestive but non-specific pattern S1Q3T3 because of right ventricular pressure overload. Right precordial leads may occasionally exhibit an ST-segment elevation [12]. Pulmonary embolism might also present with T wave inverted in either the anterior or inferior leads or perhaps both [13]. Although pulmonary embolism (PE) appearing as STEMI is an uncommon occurrence, it has been documented in several cases [14-17]. Table 1 shows the summary of case reports showing ST-segment elevation mimicking ST-segment elevation myocardial infarction (STEMI) in pulmonary embolism patients [15,18-23].

**Table 1:** Summary of case reports of pulmonary embolism mimicking ST-segment elevation myocardial infarction (STEMI) aVF: augmented vector foot The table was made by the authors of this article

Study	Age	Sex	Clinical presentation	ST-segment elevation	Coronary angiography	Cardiac enzymes
Zelfani et al., 2019 [19]	37	Female	Chest pain	V2 and V3	No significant stenosis	-
Emren et al., 2014 [20]	69	Male	Chest pain and sweating	II, III, and aVF	No significant stenosis	-
Goslar and Podbregar, 2010 [15]	57	Male	Chest pain, nausea, fatigue, and difficulty in breathing	V1-V4	No significant stenosis	Slightly elevated
Livaditis et al., 2004 [18]	42	Female	Acute, painful right leg swelling	V1-V3	No significant stenosis	Not elevated
Lin et al., 2009 [21]	35	Male	Severe chest pain and dyspnea	V1-V4	No significant stenosis	Not elevated
Paparoupa et al., 2021 [22]	80	Female	Acute dyspnea and sinus tachycardia	II, III, aVF, and V3-V6	Not performed	Elevated
Zheng et al., 2021 [23]	58	Female	Dyspnea	V1-V4	Significant stenosis	Elevated

Lung Cancer

Lung metastasis can also cause ST-segment elevation in patients by causing myocardial metastasis. In one case report, a patient with squamous cell lung cancer was diagnosed six months before showing up at the hospital with chest discomfort and dyspnea. An electrocardiogram showed ST-segment elevation in inferior and lateral leads. Even after performing percutaneous coronary intervention (PCI) on the seventh day, ECG showed persistent ST-segment elevation. Elevated ST segment can be a non-specific diagnostic test for cardiac metastasis in patients with lung cancer [24]. Another case mimicking ST-segment elevation myocardial infarction (STEMI) in a patient with lung carcinoma turned out to have pericarditis instead of myocardial infarction (MI). ECG finding shows ST-segment elevation at leads II and aVF; there was neither reciprocal ST depression nor Q wave evolution in this patient at lead I or augmented vector left (aVL). So, this clinical condition known as regional pericarditis can mimic STEMI [25].

Pneumothorax

Pneumothorax is a condition in which the air or gas accumulation in the pleural cavity might cause difficulty in breathing. The relation between tension pneumothorax and ST-segment elevation can be seen in precordial leads masquerading ST-segment elevation as myocardial infarction. The mechanism of ST-segment elevation is that a hypertensive pneumothorax has the capacity to cause hypotension, which lowers coronary blood flow, therefore leading to ischemia of myocardial tissues, which causes ECG changes [4]. In approximately 25% of the cases of pneumothorax, abnormal ECG can be seen [26]. One case report presented a patient with right tension pneumothorax with STEMI-like ST-segment increase in leads I, II, III, and aVF and ST-segment depression in leads I, aVL, and V2-V5. After treating the patient for pneumothorax, a chest tube was placed, and one hour later, the abnormality of the ST segment became normal. The authors speculate that abnormalities in the ST segment were because of the compression of the heart or right coronary artery mimicking STEMI in ECG, but the complete mechanism is still unknown [27].

Gastrointestinal system

Hiatal Hernia

In a hiatal hernia, a gap in the diaphragm allows the top portion of the stomach or another internal organ to protrude out of the diaphragm into the chest cavity. The muscular diaphragm helps with breathing and has a narrow opening called a hiatus through which the esophagus travels before joining with the stomach. The gastroesophageal junction is also known as GEJ. A hiatal hernia weakens the lower esophageal sphincter (LES) when the stomach makes its way through the opening into the chest. This laxity of the LES, which is the main cause of gastroesophageal reflux disease (GERD), can result in stomach contents and acid backing up into the esophagus (GERD). Large hiatal hernias require surgical repair, while small ones are often asymptomatic and can be treated medically [28,29]. Hiatal hernias can be inherited or acquired [30]. The compression of the heart chambers from hiatus hernias can result in exertional dyspnea, aberrant electrocardiograms with ST-segment elevation mimicking myocardial Infarction, and elevated serum biomarkers of the acute coronary syndrome [31]. So differential must be considered in ST-segment elevation other than myocardial infarction. Hiatal hernia causes acute coronary syndrome, which has common symptoms such as dyspnea mimicking angina. Another theory suggests that hiatal hernia alters the electrocardiogram (ECG) by compressing the vagal innervation of the heart [32]. Lastly, one more mechanism suggests that pericardial irritation caused by hiatal hernia may be the cause of ECG abnormalities [33]. After surgery to treat the hiatal hernia, the ST-segment elevation fades.

Small Bowel Obstruction

Small bowel obstruction (SBO) is a critical clinical condition with high chances of complex complications such as strangulation of the bowel. Early diagnosis is essential for small bowel obstruction as it is an emergency condition. Stomach pain, nausea, vomiting, and abdominal distention are hallmarks and symptoms of the condition [34]. On 12 leads, ECG ST-segment elevation has been documented in several cases of SBO [35-38]. The explanation of ST-segment elevation in patients with SBO is believed to be that the intra-abdominal distension caused the heart's diaphragmatic surface to compress, which then caused the ensuing ECG abnormalities [36]. An alternate explanation is that the distension of the gastrointestinal tract could increase vagal tone, trigger the vasovagal reflex, and disrupt ventricular depolarization as a result [39].

In almost all cases, ST-segment elevation got resolved after surgical decompression of intestinal distension. For epigastric pain, abdominal distention, and ST-segment elevation mimicking MI, gastrointestinal pathologies should be considered as a differential diagnosis before performing invasive procedures such as coronary catheterization.

Acute Pancreatitis

The symptoms and signs of acute pancreatitis can mimic those of acute coronary syndrome (ACS), including epigastric or chest discomfort (angina), nausea, vomiting, and syncope. This makes the diagnosis more difficult when there is ST-segment elevation and a worry about ischemia [40]. Minor ECG changes that can be produced by pancreatitis can be a T wave inversion, ST-segment depression, and ST-segment elevation in the absence of underlying cardiac pathology [41]. Possible mechanisms that could cause ECG abnormalities are electrolyte disorders that can alter the repolarization phase, including hypokalemia, hypomagnesemia, hypocalcemia, and hyponatremia, which are characterized by acute pancreatitis [42]. Another mechanism is cardiac myocytes that sustain direct injury as a result of proteolytic enzymes such as trypsin. This could alter the membrane's permeability, directly harm the membrane, and cause necrosis, causing an electrical disruption and irregularities in the electrocardiogram [43,44]. Although ST-segment elevation is a rare phenomenon in the case of acute pancreatitis, it should not be ignored to avoid unnecessary cardiac catheterization and hospital care costs.

Gastric Perforation

Gastric perforation means a full-thickness injury to the organ's wall that results in a perforation of the stomach. The peritoneum entirely encircles the stomach, with a hole in the wall allowing contact between the peritoneal cavity and gastric lumen. When the hole develops rapidly, the gastric contents freely enter the general intraperitoneal space because there is no opportunity for an inflammatory response to shut off the perforation. This results in chemical peritonitis [45]. Gastric perforation is mostly caused by peptic ulcer diseases and is known as peptic ulcer perforation; other etiologies such as trauma, cancer, interventional treatments, and intrinsic gastrointestinal pathophysiology are all possibilities [46]. When a patient has ST-segment elevation, ST-segment elevation myocardial infarction (STEMI) should always be considered a possibility. The differential diagnosis for a case with the involvement of abdominal signs and symptoms should include gastric perforation or other abdominal disorders. Patients undergoing gastric perforation surgery showed normalization of ECG changes (ST-segment elevation) [47]. Cases that reported of gastric perforation mimicking STEMI mainly showed symptoms such as chest pain, dyspnea and epigastric pain, and cardiac markers that are not elevated. However, very few have reported gastric perforation mimicking STEMI, but it cannot be ignored. The shift in heart posture caused by the compressive effect of abdominal distention was the pathogenesis of ST-segment elevation in gastric perforation patients [47,48].

Esophageal Rupture

The spontaneous esophageal rupture was first reported and described by Herman Boerhaave in 1724; that is why it is also known as Boerhaave's syndrome. With fatality rates as high as 40%, Boerhaave's syndrome is one of the most lethal gastrointestinal illnesses. Variable symptoms can make diagnosis difficult. The high morbidity of the illness is also a result of a number of circumstances, including the difficulty in evaluating the esophagus and the peculiar organ blood supply. In the absence of treatment, Boerhaave's syndrome patients may only survive a few days [49-51]. The condition may show vague symptoms, or the traditional Mackler triad of vomiting, discomfort in the chest, and subcutaneous emphysema may be present. The results are better the earlier the diagnosis is made [52]. Boerhaave's syndrome can mimic many conditions, including myocardial infarction, pneumothorax, and pancreatitis [53]. Both myocardial infarction and Boerhaave's syndrome are medical emergencies that need to be diagnosed and treated as soon as possible. Few cases have been reported causing ST-segment elevation and making the diagnosis more complicated [54-57]. One thing that is persistent in all the cases was that cardiac biomarkers were negative. In descending necrotizing mediastinitis, a similar condition has been described; the ST-segment elevation mimicking STEMI is observed to be a result of the inflammation of the mediastinum [58].

Endocrine disorders

Pheochromocytoma

**Figure 1:**
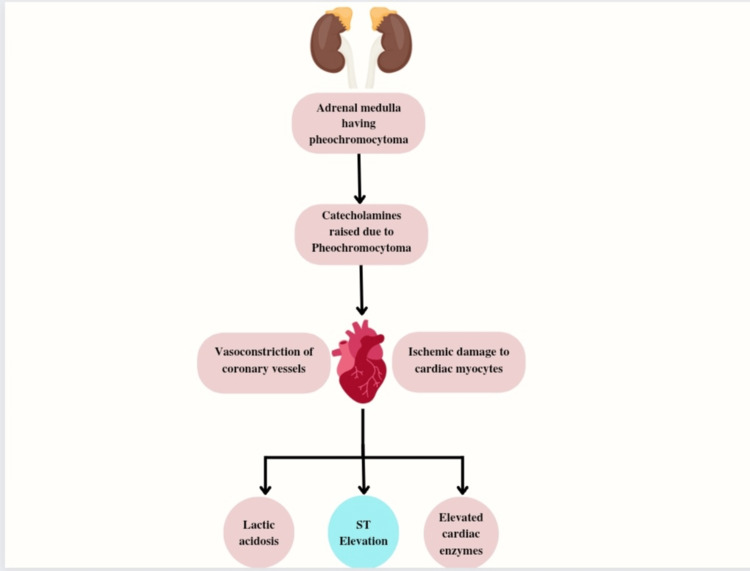
Physiology behind ST-segment elevation associated with pheochromocytoma The figure was made by the authors of this article

Pheochromocytomas are rare, often benign tumors that produce catecholamines from the adrenal medulla's chromaffin cells. Persistent or paroxysmal hypertension, excruciating headaches, palpitations, and excessive sweating are typical clinical symptoms. They can mimic many other diseases though, and their presence is extremely diverse. However, they manifest in a wide variety of ways and can resemble many different illnesses [59]. However, excessive catecholamine secretion has a noxious effect on cardiac myocytes and can restrict blood flow to the coronary arteries causing vasoconstriction and a decrease in the diameter of blood vessel [60]. Ischemic damage caused by catecholamine-induced vasoconstriction can involve several organs and result in lactic acidosis and elevated cardiac enzymes, among other indicators [61]. Pheochromocytoma and STEMI instances have been previously recorded and indicated [62,63], while a number of other cases have acknowledged a connection between pheochromocytoma and non-STEMI acute coronary syndrome [64,65]. The ramifications of this rare cardiac manifestation of pheochromocytoma should prompt a conversation about when to investigate pheochromocytoma in those who have primary coronary spasms that contribute to the sudden coronary syndrome. In normal coronary artery angiograms in the presence of acute cardiac ischemic and ST-segment elevation in an ECG, this situation should raise the possibility of additional disorders such as pheochromocytoma in the patient and should be screened for this condition so that treatment would not be delayed as it is a time-sensitive disorder [66]. Figure 1 shows the pathophysiology behind ST-segment elevation associated with pheochromocytoma.

Thyrotoxicosis

Thyrotoxicosis refers to thyroid hormone surpluses with or without elevated thyroid hormone production (hyperthyroidism). Most cases of thyrotoxicosis are linked with Graves' disease and toxic nodular goiter [67]. Hyperthyroidism is widely documented to be linked to cardiovascular disease [68]. Fatal disorders such as acute myocardial infraction presentation might be present in patients suffering from thyrotoxicosis, as seen in one case report, in which the patient presented with elevated ST segments in leads II, III, and aVF, as well as pressure-like chest discomfort and an increased troponin level. Despite the apparent lack of substantial cardiac risk factors, the presentation suggested an acute myocardial infarction (MI) [69]. Cardiovascular events are 2.6 times more likely to occur [70]. ST-segment elevation mimicking acute myocardial infarction in thyrotoxicosis is the most possible mechanism, which is believed to be caused by coronary vasospasm due to thyrotoxicosis presenting with typical angina [69]. Vasospastic angina caused by transient coronary vasospasm affects up to 20% of thyrotoxicosis patients but is difficult to detect [71]. Thyrotoxicosis-induced vasospasm may be caused by increased coronary sensitivity to vasoconstrictors and decreased sensitivity to vasodilators [72].

Electrolyte disorders

Hypocalcemia

Hypocalcemia is a potentially fatal metabolic condition that raises the possibility of critical flaws in diagnosis and therapy [73]. The most common cause of hypocalcemia is found to be vitamin D deficiency [74]. Electrolyte imbalances are the cause of electrocardiographic abnormalities. Hypocalcemia is characterized by the ST segment and corrected QT (QTc) lengthening as a result of a decrease in the phase two of the action potential. T waves can be depressed or reversed, although they generally retain their polarity. Although it is uncommon, hypocalcemia can cause ST-segment elevation [75]. After searching the PubMed and Google Scholar databases for hypocalcemia mimicking acute STEMI, many cases were found [75-79].

There is currently no ideal explanation for hypocalcemia-related ventricular contractility impairment because calcium is a critical electrolyte involved in the formation of action potentials and the contraction of heart muscle cells. A significant drop in its levels could have caused irregularities in both electrical and contractile activity, explaining those findings, as well as the lack of a relationship between regional wall motion difficulties and ST. Experimental research suggests a relationship between ventricular function depression and low calcium concentrations [80]. Another theory of "hypocalcemic cardiomyopathy" suggests that a severe fall in calcium levels might be the temporary cause of myocardial spasms and heart failure [81]. Finally, hypocalcemia can result in a "pseudo-STEMI" pattern, most commonly in the lateral leads.

Hyperkalemia

A major cause of electrolyte-induced cardiac conduction disruption is hyperkalemia, which can cause ECG changes such as QT interval shortening, T wave peaking, QRS extension, PR interval shortening, P wave amplitude decrease, the loss of sinoatrial conduction with the development of a wide-complex "sine wave" ventricular beat, asystole, and also ST-segment elevation [82]. Potassium is an essential electrolyte in both particular and non-specific cardiac tissue. As a result, significant changes in its level in plasma can have a major impact on electrical activities and can induce arrhythmias, and hyperkalemia most commonly causes an ST-segment increase in the right precordial leads, which mimics conditions such as acute myocardial infarction [83]. Physiologically, the mechanism for the ECG changes in the rise of potassium levels, and the time span of the action potential shortens and gradually moves toward less negative values [84]. Hyperkalemia-causing coronary spasms must also be evaluated [85]. The early diagnosis and management of hyperkalemia are based on the physician's ability to spot hyperkalemia-related ECG alterations; however, ECG alone is not accurate for diagnosis [86].

Central nervous system

Subarachnoid Hemorrhage (SAH)

Electrocardiographic changes are not uncommon in conditions of cerebrovascular disorders such as subarachnoid hemorrhage, and they can rarely present abnormalities in ECG, including ST-segment elevation or depression [87]. Many times, subarachnoid hemorrhage is misrepresented as acute ST-segment elevation myocardial infarction and ischemic heart disease and leads to incorrect treatment such as percutaneous coronary intervention and thrombolytic therapy, which can cause harmful effects. To prevent it, physicians must also consider cerebrovascular diseases in a patient showing ST-segment elevation mimicking STEMI.

SAH patients also have increased troponin levels and atypical cardiac regional wall motion due to neurogenic paralyzed myocardium [88]. Increased catecholamine discharge from localized nerve endings in the heart may mediate cardiac abnormalities. Transient severe coronary vascular constriction causes ischemia, which is accompanied by postischemic ventricular failure and subendocardial myocardial damage. Furthermore, catecholamine's direct cardiotoxic impact may lead to the onset of endocardial damage [88,89].

**Figure 2:**
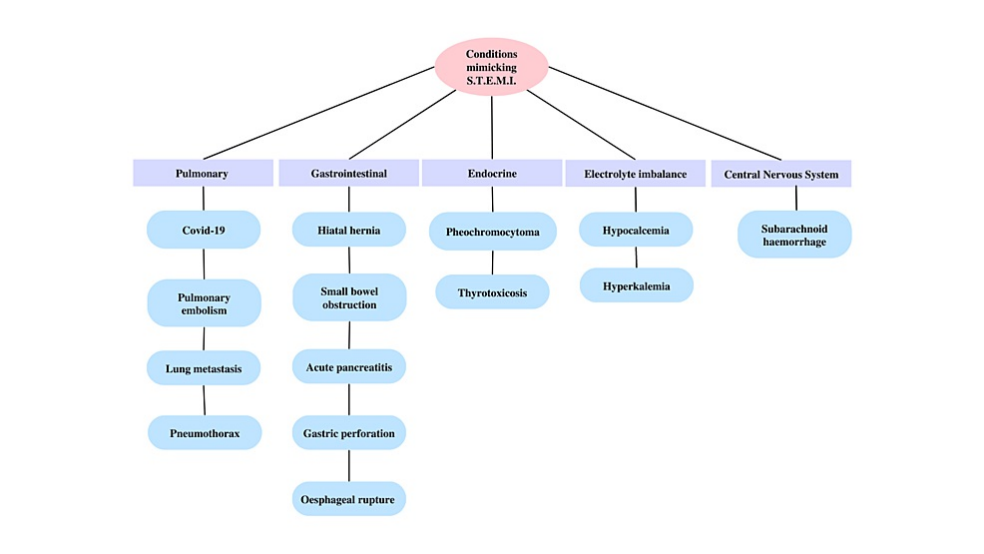
Conditions mimicking ST-segment elevation myocardial infarction (STEMI) other than cardiovascular disorders The figure was made by the authors of this article

Performing a computed tomography (CT) scan of the brain is usually recommended before the commencement of treatment with antithrombotic drugs, as it is an important therapy for acute MI [90]. So, when a patient presents with severe headache and sustained loss of consciousness with electrocardiograms showing ST-segment elevation or depression indicative of acute myocardial infarction, it should not be ignored since these may be manifestations of several neurological illnesses such as subarachnoid hemorrhage linked with stress cardiomyopathy [91]. Figure 2 shows the summary of conditions mimicking ST-segment elevation myocardial infarction (STEMI) other than cardiovascular disorders.

## Conclusions

As ECG is the most common investigation performed by emergency doctors, it is important to know different diagnoses in which ECG changes indicate various cardiac manifestations such as acute myocardial infarction and acute coronary syndrome. The most common ECG abnormality seen is ST-segment elevation mimicking STEMI. Cardiac markers might also be elevated in a few non-cardiac conditions showing ST-segment elevation, which might confuse the physician leading them toward misdiagnosis and rendering a treatment directed toward cardiovascular therapy in a non-cardiovascular comorbid patient. Due to this, the physician might prescribe clinical procedures such as coronary catheterization to the patient, which are not only unnecessary but also economically taxing and fatal.

Hence, the purpose of this narrative review was to make a brief account of several clinical conditions that present with ST-segment elevation apart from the cardiovascular disorder so that physicians can obtain a better understanding of cases that display chest pain along with ST-segment elevation but turn out to be a clinical sign of a disease involving some other systems of the body.

## References

[1] Sattar Y, Chhabra L (2022). Electrocardiogram. https://www.ncbi.nlm.nih.gov/books/NBK549803/.

[2] Akbar H, Foth C, Kahloon RA, Mountfort S (2022). Acute ST elevation myocardial infarction. https://www.ncbi.nlm.nih.gov/books/NBK532281/.

[3] Canto JG, Kiefe CI, Rogers WJ (2011). Number of coronary heart disease risk factors and mortality in patients with first myocardial infarction. JAMA.

[4] Coppola G, Carità P, Corrado E (2013). ST segment elevations: always a marker of acute myocardial infarction?. Indian Heart J.

[5] Zhou P, Yang XL, Wang XG (2020). A pneumonia outbreak associated with a new coronavirus of probable bat origin. Nature.

[6] Nishiga M, Wang DW, Han Y, Lewis DB, Wu JC (2020). COVID-19 and cardiovascular disease: from basic mechanisms to clinical perspectives. Nat Rev Cardiol.

[7] Mahmud E, Dauerman HL, Welt FG (2020). Management of acute myocardial infarction during the COVID-19 pandemic: a consensus statement from the Society for Cardiovascular Angiography and Interventions (SCAI), the American College of Cardiology (ACC), and the American College of Emergency Physicians (ACEP). Catheter Cardiovasc Interv.

[8] Diaz-Arocutipa C, Torres-Valencia J, Saucedo-Chinchay J, Cuevas C (2021). ST-segment elevation in patients with COVID-19: a systematic review. J Thromb Thrombolysis.

[9] Bhandari B, Neupane S, Khanal R, Lnu K, Wert Y, Komanduri S (2021). COVID-19 pericarditis mimicking an acute myocardial infarction: a case report and review of literature. J Community Hosp Intern Med Perspect.

[10] Arrigo M, Huber LC (2020). Pulmonary embolism and heart failure: a reappraisal. Card Fail Rev.

[11] Torbicki A, Perrier A, Konstantinides S (2008). Guidelines on the diagnosis and management of acute pulmonary embolism: the Task Force for the Diagnosis and Management of Acute Pulmonary Embolism of the European Society of Cardiology (ESC). Eur Heart J.

[12] Kosuge M, Kimura K, Ishikawa T (2007). Electrocardiographic differentiation between acute pulmonary embolism and acute coronary syndromes on the basis of negative T waves. Am J Cardiol.

[13] Hanna EB, Glancy DL (2015). ST-segment elevation: differential diagnosis, caveats. Cleve Clin J Med.

[14] Dasa O, Ruzieh M, Ammari Z, Syed MA, Brickman KR, Gupta R (2018). It's a ST-elevation myocardial infarction (STEMI), or is it? Massive pulmonary embolism presenting as STEMI. J Emerg Med.

[15] Goslar T, Podbregar M (2010). Acute ECG ST-segment elevation mimicking myocardial infarction in a patient with pulmonary embolism. Cardiovasc Ultrasound.

[16] Villablanca PA, Vlismas PP, Aleksandrovich T (2019). Case report and systematic review of pulmonary embolism mimicking ST-elevation myocardial infarction. Vascular.

[17] Özer N, Yorgun H, Canpolat U, Ateş AH, Aksöyek S (2011). Pulmonary embolism presenting with evolving electrocardiographic abnormalities mimicking anteroseptal myocardial infarction: a case report. Med Princ Pract.

[18] Livaditis IG, Paraschos M, Dimopoulos K (2004). Massive pulmonary embolism with ST elevation in leads V1-V3 and successful thrombolysis with tenecteplase. Heart.

[19] Zelfani S, Manai H, Laabidi S, Wahabi A, Akeri S, Daghfous M (2019). Pulmonary embolism mimicking acute myocardial infarction: a case report and review of literature. Pan Afr Med J.

[20] Emren SV, Arıkan ME, Senöz O, Varış E, Akan E (2014). Acute pulmonary embolism mimicking inferior myocardial infarction. Turk Kardiyol Dern Ars.

[21] Lin JF, Li YC, Yang PL (2009). A case of massive pulmonary embolism with ST elevation in leads V1-4. Circ J.

[22] Paparoupa M, Aldemyati R, Theodorakopoulou M (2021). Bilateral lung artery embolization mimicking an acute myocardial infarction. Case Rep Med.

[23] Zheng B, Bian F, Li J, Xu H, Wang J (2022). A potential diagnostic pitfall in ST elevation: acute pulmonary embolism or ST-segment elevation myocardial infarction. Ann Noninvasive Electrocardiol.

[24] Jung HW (2021). ST-segment elevation due to myocardial invasion of lung cancer mimicking ST elevation myocardial infarction: a case report. Medicine (Baltimore).

[25] Bijay Y, Miriyala V, Pant S (2021). Lung carcinoma paraneoplastic reactive regional pericarditis mimicking ST elevation myocardial infarct. J Community Hosp Intern Med Perspect.

[26] Krenke R, Nasilowski J, Przybylowski T, Chazan R (2008). Electrocardiographic changes in patients with spontaneous pneumothorax. J Physiol Pharmacol.

[27] Kataoka E, Kimura M, Iwai T, Sawada T (2022). ST-segment elevation mimicking inferior wall myocardial infarction caused by right tension pneumothorax. Circ J.

[28] Kahrilas PJ, Kim HC, Pandolfino JE (2008). Approaches to the diagnosis and grading of hiatal hernia. Best Pract Res Clin Gastroenterol.

[29] Smith RE, Shahjehan RD (2022). Hiatal hernia. https://www.ncbi.nlm.nih.gov/books/NBK562200/.

[30] Hyun JJ, Bak YT (2011). Clinical significance of hiatal hernia. Gut Liver.

[31] Rubini Gimenez M, Gonzalez Jurka L, Zellweger MJ, Haaf P (2019). A case report of a giant hiatal hernia mimicking an ST-elevation myocardial infarction. Eur Heart J Case Rep.

[32] Schilling RJ, Kaye GC (1998). Paroxysmal atrial flutter suppressed by repair of a large paraesophageal hernia. Pacing Clin Electrophysiol.

[33] Hokamaki J, Kawano H, Miyamoto S (2005). Dynamic electrocardiographic changes due to cardiac compression by a giant hiatal hernia. Intern Med.

[34] Taylor MR, Lalani N (2013). Adult small bowel obstruction. Acad Emerg Med.

[35] Parikh M, Amor MM, Verma I, Osofsky J, Paladugu M (2015). Small bowel obstruction masquerading as acute ST elevation myocardial infarction. Case Rep Cardiol.

[36] Patel K, Chang NL, Shulik O, DePasquale J, Shamoon F (2015). Small bowel obstruction mimicking acute ST-elevation myocardial infarction. Case Rep Surg.

[37] Upadhyay A, Chauhan S, Jangda U, Bodar V, Al-Chalabi A (2017). Reversible inferolateral ST-segment elevation associated with small bowel obstruction. Case Rep Med.

[38] Banerjee R, Hillerson D, Leung SW, Sorrell VL (2021). ST-segment elevation in a patient with nausea, vomiting, and intracerebral hemorrhage. JACC Case Rep.

[39] Mixon TA, Houck PD (2003). Intestinal obstruction mimicking acute myocardial infarction. Tex Heart Inst J.

[40] Agrawal A, Sayyida N, Penalver JL, Ziccardi MR (2018). Acute pancreatitis mimicking ST-segment elevation myocardial infarction. Case Rep Cardiol.

[41] Patel J, Movahed A, Reeves WC (1994). Electrocardiographic and segmental wall motion abnormalities in pancreatitis mimicking myocardial infarction. Clin Cardiol.

[42] Chou TC (1973). Pseudo-infarction (noninfarction Q waves). Cardiovasc Clin.

[43] Franzen D, Jung S, Fatio R, Brunckhorst CB (2009). Complete atrioventricular block in a patient with acute cholecystitis: a case of cardio-biliary reflex?. Eur J Emerg Med.

[44] Saulea A, Costin S, Rotari V (1997). Heart ultrastructure in experimental acute pancreatitis. Rom J Physiol.

[45] Sigmon DF, Tuma F, Kamel BG, Cassaro S (2022). Gastric perforation. https://www.ncbi.nlm.nih.gov/books/NBK519554/.

[46] Chen TY, Liu HK, Yang MC (2018). Neonatal gastric perforation: a report of two cases and a systematic review. Medicine (Baltimore).

[47] Intan RE, Hasibuan FS, Gandi P, Alkaff FF (2021). Gastric perforation mimicking ST-segment elevation myocardial infarction. BMJ Case Rep.

[48] Herath HM, Thushara Matthias A, Keragala BS, Udeshika WA, Kulatunga A (2016). Gastric dilatation and intestinal obstruction mimicking acute coronary syndrome with dynamic electrocardiographic changes. BMC Cardiovasc Disord.

[49] Cuccì M, Caputo F, Fraternali Orcioni G, Roncallo A, Ventura F (2018). Transition of a Mallory-Weiss syndrome to a Boerhaave syndrome confirmed by anamnestic, necroscopic, and autopsy data: a case report. Medicine (Baltimore).

[50] He F, Dai M, Zhou J, He J, Ye B (2018). Endoscopic repair of spontaneous esophageal rupture during gastroscopy: a CARE compliant case report. Medicine (Baltimore).

[51] Ciriano Hernández P, Grao Torrente I, Viejo Martínez E, Turégano Fuentes F (2019). Boerhaave syndrome presenting as gastric emphysema. Cir Esp (Engl Ed).

[52] Turner AR, Turner SD (2022). Boerhaave syndrome. https://www.ncbi.nlm.nih.gov/books/NBK430808/.

[53] Vidarsdottir H, Blondal S, Alfredsson H, Geirsson A, Gudbjartsson T (2010). Oesophageal perforations in Iceland: a whole population study on incidence, aetiology and surgical outcome. Thorac Cardiovasc Surg.

[54] Shemesh A, Taub CC (2016). Inferolateral ST-segment elevation in Boerhaave syndrome. Am J Med.

[55] Skaug B, Taylor KR, Chandrasekaran S (2016). Oesophageal rupture masquerading as STEMI. BMJ Case Rep.

[56] Rigger W, Mai R, Maddux PT, Cavalieri S, Calkins J (2021). Esophageal rupture presenting with ST segment elevation and junctional rhythm mimicking acute myocardial infarction. Case Rep Crit Care.

[57] Inci S, Gundogdu F, Gungor H, Arslan S, Turkyilmaz A, Eroglu A (2013). Misdiagnosed chest pain: spontaneous esophageal rupture. Acta Cardiol Sin.

[58] Ochi N, Wakabayashi T, Urakami A (2018). Descending necrotizing mediastinitis in a healthy young adult. Ther Clin Risk Manag.

[59] Reisch N, Peczkowska M, Januszewicz A, Neumann HP (2006). Pheochromocytoma: presentation, diagnosis and treatment. J Hypertens.

[60] Gu YW, Poste J, Kunal M, Schwarcz M, Weiss I (2017). Cardiovascular manifestations of pheochromocytoma. Cardiol Rev.

[61] Manger WM (2006). An overview of pheochromocytoma: history, current concepts, vagaries, and diagnostic challenges. Ann N Y Acad Sci.

[62] Beedupalli J, Akkus NI (2014). Concealed pheochromocytoma presenting as recurrent acute coronary syndrome with STEMI : case report of a patient with hyperthyroidism. Herz.

[63] Hayıroğlu Mİ, Yıldırımtürk Ö, Bozbay M, Eren M, Pehlivanoğlu S (2015). Hypertensive emergency due to pheochromocytoma crisis complicated with refractory hemodynamic collapse. Turk Kardiyol Dern Ars.

[64] Lee TW, Lin KH, Chang CJ, Lew WH, Lee TI (2013). Pheochromocytoma mimicking both acute coronary syndrome and sepsis: a case report. Med Princ Pract.

[65] Horton WB, Frey LM, Hawkins UA, Ahmad SR (2015). Pheochromocytoma presenting as acute non-ST elevation myocardial infarction following elective hysterectomy. J Miss State Med Assoc.

[66] Ahmed MA, Abdullah AS, Kiernan TJ (2016). Phaeochromocytoma presenting with ST segment elevation myocardial infarction. BMJ Case Rep.

[67] Gilbert J (2017). Thyrotoxicosis - investigation and management. Clin Med (Lond).

[68] Krishnan GD, Yahaya N, Yahya M (2019). Hyperthyroidism presenting as ST elevation myocardial infarction with normal coronaries - a case report. J ASEAN Fed Endocr Soc.

[69] Rymer De Marchena I, Gutman A, Zaidan J, Yacoub H, Hoyek W (2017). Thyrotoxicosis mimicking ST elevation myocardial infarction. Cureus.

[70] Zhou D, Qu Z, Wang H, Wang Z, Xu Q (2015). Severe hyperthyroidism presenting with acute ST segment elevation myocardial infarction. Case Rep Cardiol.

[71] Dedov II, Kalashnikov VIu, Terekhin SA (2012). [Fatal coronary artery spasm in a patient with thyrotoxicosis] [Article in Russian]. Kardiologiia.

[72] Kohno A, Hara Y (2001). Severe myocardial ischemia following hormone replacement in two cases of hypothyroidism with normal coronary arteriogram. Endocr J.

[73] Cooper MS, Gittoes NJ (2008). Diagnosis and management of hypocalcaemia. BMJ.

[74] Holick MF (2007). Vitamin D deficiency. N Engl J Med.

[75] Peiro B, Cerdán L, Diarte JA, Ortas MR, Cortés C (2022). Severe hypocalcemia mimicking acute ST-segment elevation myocardial infarction: paradigmatic case and review of literature. Cardiol J.

[76] Adeel MY, Clarke JD, Shetty S, Arora A, Buscher MG (2018). Severe hypocalcemia mimicking acute inferior ST-segment elevation myocardial infarction. Oxf Med Case Reports.

[77] Kukla P, Kulik M, Jastrzębski M, Bryniarski L, Czarnecka D, Baranchuk A (2017). Severe hypocalcemia mimicking ST-segment elevation acute myocardial infarction. Ann Noninvasive Electrocardiol.

[78] Lehmann G, Deisenhofer I, Ndrepepa G, Schmitt C (2000). ECG changes in a 25-year-old woman with hypocalcemia due to hypoparathyroidism. Hypocalcemia mimicking acute myocardial infarction. Chest.

[79] Ilveskoski E, Sclarovsky S, Nikus K (2012). Severe hypocalcemia simulating ST-elevation myocardial infarction. Am J Emerg Med.

[80] Lang RM, Fellner SK, Neumann A, Bushinsky DA, Borow KM (1988). Left ventricular contractility varies directly with blood ionized calcium. Ann Intern Med.

[81] Aguiar P, Cruz D, Ferro Rodrigues R, Peixoto L, Araújo F, Ducla Soares JL (2013). Hypocalcemic cardiomyopathy. Rev Port Cardiol.

[82] Weiss JN, Qu Z, Shivkumar K (2017). Electrophysiology of hypokalemia and hyperkalemia. Circ Arrhythm Electrophysiol.

[83] Sims DB, Sperling LS (2005). Images in cardiovascular medicine. ST-segment elevation resulting from hyperkalemia. Circulation.

[84] Levine HD, Wanzer SH, Merrill JP (1956). Dialyzable currents of injury in potassium intoxication resembling acute myocardial infarction or pericarditis. Circulation.

[85] Pastor JA, Castellanos A, Moleiro F, Myerburg RJ (2001). Patterns of acute inferior wall myocardial infarction caused by hyperkalemia. J Electrocardiol.

[86] Wrenn KD, Slovis CM, Slovis BS (1991). The ability of physicians to predict hyperkalemia from the ECG. Ann Emerg Med.

[87] Zaroff JG, Rordorf GA, Newell JB, Ogilvy CS, Levinson JR (1999). Cardiac outcome in patients with subarachnoid hemorrhage and electrocardiographic abnormalities. Neurosurgery.

[88] Nguyen H, Zaroff JG (2009). Neurogenic stunned myocardium. Curr Neurol Neurosci Rep.

[89] Yuki K, Kodama Y, Onda J, Emoto K, Morimoto T, Uozumi T (1991). Coronary vasospasm following subarachnoid hemorrhage as a cause of stunned myocardium. Case report. J Neurosurg.

[90] Lee HC, Yen HW, Lu YH, Lee KT, Voon WC, Lai WT, Sheu SH (2004). A case of subarahnoid hemorrhage with persistent shock and transient ST elevation simulating acute myocardial infarction. Kaohsiung J Med Sci.

[91] Enache I, Radu RA, Terecoasă EO, Dorobăţ B, Tiu C (2020). Stress cardiomyopathy misinterpreted as ST-segment elevation myocardial infarction in a patient with aneurysmal subarachnoid hemorrhage: a case report. Rom J Intern Med.

